# The Axl-Regulating Tumor Suppressor miR-34a Is Increased in ccRCC but Does Not Correlate with Axl mRNA or Axl Protein Levels

**DOI:** 10.1371/journal.pone.0135991

**Published:** 2015-08-19

**Authors:** Helena K. Fritz, Anna Gustafsson, Börje Ljungberg, Yvonne Ceder, Håkan Axelson, Björn Dahlbäck

**Affiliations:** 1 Lund University, Department of Translational Medicine, Section of Clinical Chemistry, University Hospital Malmö, Malmö, Sweden; 2 Umeå University, Departments of Surgical and Perioperative Sciences, Urology and Andrology, Umeå, Sweden; 3 Lund University, Department of Laboratory Medicine, Division of Translational Cancer Research, Medicon Village, Lund, Sweden; Univeristy of California Riverside, UNITED STATES

## Abstract

**Background:**

High expression of the receptor tyrosine kinase Axl is associated with poor prognosis in patients with Renal Cell Carcinoma (RCC), the most common malignancy of the kidney. The miR-34a has been shown to directly regulate Axl in cancer cells. The miR-34a is a mediator of p53-dependent tumor suppression, and low expression of miR-34a has been associated with worse prognosis in several cancers. Our aim was to elucidate whether miR-34a or the other members of the miR-34 family (miR-34b/c) regulate Axl in RCC.

**Methodology and Results:**

Using western blot, flow cytometry, and RT-qPCR, we showed that Axl mRNA and protein are downregulated in 786-O cells by miR-34a and miR-34c but not by miR-34b. A luciferase reporter assay demonstrated direct interaction between the Axl 3’ UTR and miR-34a and miR-34c. The levels of miR-34a/b/c were measured in tumor tissue in a cohort of 198 RCC patients, and the levels of miR-34a were found to be upregulated in clear cell RCC (ccRCC) tumors, but not associated with patient outcome. Neither of the miR-34 family members correlated with Axl mRNA, soluble Axl protein in serum, nor with immunohistochemistry of Axl in tumor tissue. In addition, we measured mRNA levels of a known miR-34a target, HNF4A, and found the HNF4A levels to be decreased in ccRCC tumors, but unexpectedly correlated positively rather than negatively with miR-34a.

**Conclusions:**

Although miR-34a and miR-34c can regulate Axl expression *in vitro*, our data indicates that the miR-34 family members are not the primary regulators of Axl expression in RCC.

## Introduction

Renal Cell Carcinoma (RCC) is the most common malignancy of the kidney, causing approximately 85% of all kidney tumors [1]. RCC is resistant to both radio- and chemotherapies, while surgery remains to be the only curative treatment [2, 3]. While RCC patients diagnosed with localized disease have a 5-year survival of 69% [1], the 5-year survival for patients with metastatic RCC is approximately 10 to 12%; the main reason for the poor prognosis in the latter is acquired resistance to targeted therapies [2, 4, 5]. Approximately 15–25% of the RCC patients are diagnosed with metastatic RCC, and another 20–25% of patients develop distant metastasis despite complete surgical removal of the initial tumor [6]. The three most common subtypes of RCC are clear cell RCC (ccRCC), papillary RCC (pRCC), and chromophobe RCC (chRCC), with ccRCC accounting for 75–80% of all RCC cases [7].

Axl is a receptor tyrosine kinase (RTK) of the TAM (Tyro3, Axl and Mer) family, which is involved in the regulation of innate immunity and clearance of apoptotic cells [8]. Axl is reported to be overexpressed in a number of malignancies [8, 9], including RCC where it has been shown to predict patient outcome [10, 11]. Axl signaling involves a number of pro-tumorigenic pathways including Akt, ERK, SRC and STAT3 [12]. Recently, Axl overexpression has been linked to targeted therapy resistance in several types of cancer [9, 13, 14].

MicroRNAs (miRNAs) are single-stranded short RNA molecules, approximately 22 nucleotides long, which act as post-transcriptional regulators of target mRNA, primarily through inhibition of translation, or degradation of mRNA strands [15]. A large number of miRNA seed sequences within mRNAs are evolutionally conserved amongst mammals [16], and a single miRNA is capable of regulating hundreds of target mRNAs [17]. The outcome on proteomic level is dependent on the molecular abundance of the different target transcripts, indicating that the effect of a single miRNA can be cell type-dependent [18].

The miR-34 family comprises three miRNAs, miR-34a, miR-34b and miR-34c, having evolutionally conserved and similar nucleotide seed sequences. They are positively regulated by p53 and function as tumor suppressors by induction of cell cycle arrest, apoptosis or senescence [19]. In some cancers, including RCC, expression of miR-34 family members is inactivated through methylation of promoter region CpG islands [20], and decreased expression of miR-34c has been associated with worse outcome in prostate cancer [21]. MiR-34a has been shown to regulate Axl translation through direct binding to the 3’ UTR of the Axl mRNA transcript, and miR-34a expression is inversely correlated to the expression of Axl in breast cancer, non-small cell lung cancer, and colorectal cancer [22]. MiR-34a is reported to be downregulated in RCC cell lines [23] but in a study comprising eight patient matched-pair tumor and normal samples, miR-34a was found to be upregulated in tumor specimens [24]. In addition, miR-34a has been shown to support cellular proliferation in an experimental renal carcinogenesis model in rats [25]. Another target for miR-34a is hepatocyte nuclear factor 4 alpha (HNF4A), a tumor suppressor in hepatocytes [26, 27]. HNF4A expression has been reported to be decreased in RCC tumors [28].

In this report, we evaluate the role of the miR-34 family members in the regulation of Axl in RCC and show that *in vitro* both miR-34a and miR-34c regulate Axl expression through direct binding to the Axl 3’UTR. In clinical RCCs, miR-34a expression was found to be increased, but did not correlate with Axl expression. In addition, we confirm that HNF4A expression is decreased in ccRCC tumors but unexpectedly found that the miR-34a levels correlated positively rather than negatively with HNF4A expression.

## Materials and Methods

### Cell culture

Clear cell renal adenocarcinoma 786-O cells were purchased from ATCC (Cat no. CRL-1932, LGC standards, Middlesex, UK). 786-O and HEK293 cells were cultured in high-glucose DMEM media (Gibco/Thermo Fisher, Waltham, MA, USA) supplied with 10% FCS (Gibco/Thermo Fisher), L-glutamine (Gibco/Thermo Fisher) and Penicillin/Streptomycin (Gibco/Thermo Fisher). During transfection, Opti-MEM (Gibco/Thermo Fisher) without any supplements was used.

### Transfection with microRNA and siRNA

Cells were transiently transfected using Oligofectamine reagent (Invitrogen/Thermo Fisher) and miRIDIAN microRNA mimics (Dharmacon/GE Healthcare, Little Chalfont, UK), according to manufacturer’s recommendations. The microRNA mimics were miR-34a (MIMAT0000255), miR-34b (MIMAT0004676), and miR-34c (MIMAT0000686). For Axl knockdown, a pool of three Axl-targeting siRNA duplexes (Santa Cruz Biotechnologies, Dallas, TX, USA) was used, and a mix of two scramble siRNAs (Santa Cruz) with no homology to human mRNAs was used as negative control. Briefly, 2.5x10^5^ cells were seeded per well in a 6-well plate (Nalgene Nunc, Penfield, NY, USA) 24 hours prior to transfection, and were transiently transfected in duplicates with 200 nM microRNA mimic. Control experiments were performed in parallel, using a miRIDIAN microRNA mimic Negative Control (Dharmacon), which is unrelated to any known human miRNAs. 24 hours after transfection, cells were split 1:3 with TrypLE Express (Gibco/Thermo Fisher) into 6-well plates, for multiplexing WB and RT-qPCR experiments, and were cultured for another 48 hours before they were harvested. For flow cytometry experiments, 0.8x10^5^ cells were seeded per well in 12-well plates 24 hours in advance of transfection, and were transfected in duplicates with 200 nM microRNA mimic or siRNA as described above. All cells were harvested 72 hours after transfection, as control experiments indicated that maximal knockdown of Axl by miRNAs as well as siRNA occurred between 48 and 72 hours.

### Analysis of protein expression using flow cytometry

Cells were harvested using TrypLE Express. The cells were pre-blocked prior to staining in 1% BSA-PBS, and were stained with 10 μg/mL of goat anti-Axl polyclonal IgG antibody (Cat no. AF154, R&D systems, Minneapolis, MN, USA) or 20 μL/sample of normal goat IgG antibody (Cat no. sc-3887, Santa Cruz), and the anti-Axl or normal goat IgG was detected using 1 μL/sample of a secondary DyLight488-conjugated donkey anti-goat polyclonal antibody (Cat no. ab96931, Abcam, Cambridge, UK) according to the manufacturer’s instructions. A negative control consisting of Axl siRNA-transfected cells stained for Axl was included in addition to the normal goat IgG-stained sample. DyLight488 signals were recorded in the FL1 channel on a FC500 flow cytometer (Beckman Coulter, Brea, CA, USA), and analysis was performed using FlowJo (TreeStar, Ashland, OR, USA) software.

### Analysis of protein expression using Western Blot

Cells were harvested with M-PER reagent (Thermo Fisher) supplied with HALT Protease Inhibitor Cocktail (Pierce/Thermo Fisher), at room temperature (RT) according to the manufacturer’s instructions. The cells were scraped, cellular debris was removed by pelleting at 10000x*g*, 5 minutes at RT, and the samples were stored at -70°C until further use. Total protein content was evaluated using a BCA assay kit (Pierce/Thermo Fisher), and an equal amount of total protein from each sample was loaded onto a 4–15% TGX SDS-PAGE mini gel (Bio-Rad Laboratories, Munich, Germany), and was blotted onto a PVDF membrane using the Trans-Blot Turbo (Bio-Rad Laboratories), Mixed MW program. The WB membranes were incubated with rabbit anti-human Axl polyclonal antibody (Cat no. sc-20741, Santa Cruz) diluted 1:1000, and mouse anti-human β-Actin monoclonal antibody (Cat no. A5441, Sigma-Aldrich, St. Louis, MO, USA) diluted 1:50000, in 3% Fish gelatin in TBS-T. Signals for secondary HRP-conjugated antibodies, swine anti-rabbit polyclonal antibody (Cat no. P0339, DAKO/Agilent, Santa Clara, CA, USA) diluted 1:1000, or goat anti-mouse polyclonal antibody (Cat no. P0447, DAKO/Agilent) diluted 1:1000, were developed using Immobilon ECL reagent (Millipore, Billerica, MA, USA) and detected with a CCD camera (Bio-Rad Laboratories). Band intensities were quantified and normalized to β-Actin band intensities using ImageLab software (Bio-Rad Laboratories).

### Extraction of RNA

For RT-qPCR gene expression analysis of mRNA transcripts in transiently transfected cell lines, total RNA was extracted using the RNeasy Plus mini kit (Qiagen, Venlo, Netherlands) according to the manufacturer’s protocol using a QiaVac device (Qiagen). For determination of mature microRNA levels in renal cell lines, total RNA was extracted using the *mir*Vana miRNA isolation kit (Ambion, Austin, TX, USA), following the manufacturer’s recommended protocol. Cell line RNA samples were quantified using a NanoDrop device (Thermo Fisher), and were stored at -70°C until further use.

Cohort samples were collected immediately after nephrectomy, and total RNA was extracted from viable tumor or kidney cortex tissues using TRIzol reagent (Ambion). RNA quality control of patient samples has been described elsewhere[29].

### RT-qPCR

The mature miRNA expression levels were quantified using the TaqMan MicroRNA Assay protocol and reagents (Applied Biosystems, Foster City, CA, USA), according to the manufacturer’s protocol, scaled down to 10 μL reaction volumes. The Reverse Transcription (RT) step was run on a T100 thermal cycler (Bio-Rad Laboratories). Quantification of the mature miRNA levels was done using the comparative–*ΔΔCt* method, using the small non-coding RNA RNU48 as an internal control for cell line samples, and for cohort samples the geometric mean of U47, RNU44 and RNU48 was used as an internal control as previously determined [29, 30]. For quantification of mRNA transcripts, the SuperScript III One-step RT-PCR system kit (Invitrogen/Thermo Fisher), and hAxl-FAM or hHNF4A-FAM and hB2M-VIC probes (Applied Biosystems) were used according to the manufacturer’s recommendation, scaled down to 10 μL reaction volumes. All RT-qPCR reactions were run on a 7900HT Real-Time PCR System (Applied Biosystems), except for RT-qPCR for Axl in miRNA-transfected 786-O cells, and RT-qPCR for HNF4A in the RCC cohort, which were run on a ViiA7 Real-Time PCR system (Applied Biosystems). Along with each RT and qPCR, or RT-qPCR reaction plate, negative control samples were run. Each sample was run in quadruplicates. For cell line experiments, Axl mRNA levels were quantified using the comparative–*ΔΔCt* method with B2M as the internal control. Quantification of the HNF4A mRNA levels was done using the comparative–*ΔΔCt* method, using B2M as the internal control. The Axl mRNA levels in the RCC patient cohort were quantified using the standard curve method with B2M as the internal control, and the Axl data have previously been published with a detailed method description [10].

### pMirTarget-AxlUTR and pMirTarget-AxlMUT vectors

The pMirTarget-AxlUTR plasmid containing the full 3’ UTR of Axl transcript 1 (RefSeq: NM_021913.3) was purchased from Origene (Cat no. SC216808, Rockville, MD, USA). The QuikChange kit (Agilent) was used to introduce a mutation in the 8-nt seed sequence in the Axl 3’ UTR, according to the manufacturer’s recommendations. The primers used for mutagenesis were AxlDelSeqQC FWD (CAGGATCCAAGCTAAGCATGACCACTGGGGAAAACTCCACC, MWG, Germany) and AxlDelSeqQC REV (GGTGGAGTTTTCCCCAGTGGTCATGCTTAGCTTGGATCCTG, MWG, Germany). The plasmids were harvested using the Miniprep kit (Qiagen) and DNA content was evaluated using the NanoDrop (Thermo Fisher). To confirm successful mutation of the seed sequence, the plasmids were sequenced using the Big Dye kit (Agilent). Purification of the sequencing samples was performed using the DyeEx 2.0 spin kit (Qiagen), analyzed by the in-house DNA Lab (Malmö University Hospital, Sweden).

### Luciferase assay

The pRL Renilla luciferase vector was a kind gift from Dr. Lars Rönnstrand. Cells were transiently transfected in duplicates with miRIDIAN microRNA mimics (Dharmacon), pMirTarget-AxlUTR/-AxlMUT/-EMPTY vectors, and Renilla luciferase transfection control pRL vector, using Lipofectamine-2000 reagent (Invitrogen/Thermo Fisher), according to manufacturer’s recommendations. Briefly, 2.5x10^5^ HEK293 cells were seeded per well in 12-well plates (Nalgene Nunc) 24 hours prior to transfection, and were transiently transfected at 70–90% confluence with 200 nM microRNA mimic, 800 ng pMirTarget-AxlUTR/-AxlMUT/-EMPTY vector, and 32 ng pRL vector. 48 hours after transfection, cells were harvested and analyzed using the Dual-Glo Luciferase kit (Promega, Fitchburg, WI, USA) in a Victor2 plate reader (Perkin Elmer, Waltham, MA, USA), and each sample was tested in duplicates in the luciferase assay. Firefly luciferase activity was normalized to the Renilla luciferase activity.

### Cohort information

The RCC patient cohort comprised samples from a total of 198 patients, and 50 non-malignant kidney cortex samples of which 34 were ccRCC matched-pairs. The study was approved by the Umeå University institutional review board and ethics committee, and with the written informed consent of the participating patients. The cohort parameters and study design have been described in detail elsewhere [10, 29].

### Sequencing

For each sample, 100 ng of total RNA was reverse transcribed using the High-Capacity cDNA Reverse Transcription kit (Applied Biosystems) according to the manufacturer’s instructions, and the RT step was performed on a T100 thermal cycler (Bio-Rad Laboratories). The region of interest was amplified using AmpliTaq 360 Gold DNA polymerase (Applied Biosystems) with the following primers; AxlSeedSeq-FWD (CATCCTGCTGGACGCTATGT, MWG Germany) and AxlSeedSeq-REV (GGGATGGAGGTGGGATAGGT, MWG, Germany), on a T100 thermal cycler (Bio-Rad Laboratories). The PCR was run according to the manufacturer’s instructions, with 1 μL GC enhancer and 2 μL MgCl_2_ (final concentration: 2 mM) per 25 μL reaction, and the annealing temperature set to 61.4°C. The PCR products were purified using the ChargeSwitch-Pro PCR Clean-Up Kit (Invitrogen/Thermo Fisher), and RNA concentration was quantified using the NanoDrop device (Thermo Fisher). Finally, 30 ng of purified PCR product per sample was added to a 96-well plate along with forward (AxlSSSeq-FWD; TATGTCCTCTGCCCTTCCAC, MWG, Germany) or reverse (AxlSSSeq-REV; AGGGCTGCAAGTGGGGATAA, MWG, Germany) sequencing primer, and was sent for sequencing at Eurofins MWG (MWG, Germany).

### Statistical analysis

Cell line experiments were analyzed using Prism software (GraphPad, LaJolla, CA, USA), one-way ANOVA test with Dunnett’s multiple comparisons test. Cohort data was managed and analyzed using the SPSS software package, and figures were generated using the Prism statistical software (GraphPad, LaJolla, CA, USA). The Kruskal-Wallis multiple comparison test was used for analysis of miRNA expression levels between RCC subtypes, the Mann-Whitney test was used to compare expression levels of miRNA and HNF4A expression between ccRCC and normal samples, and Axl-miRNA and HNF4A-miRNA correlations were evaluated using Spearman correlation. For comparing cumulative survival, the Kaplan-Meier method and the Mantel-Cox Log Rank test were used. All tests were two-sided and with the significance level set to 0.05.

## Results

### Axl protein expression decreased in 786-O cells transfected with miR-34a or miR-34c

**Fig 1:**
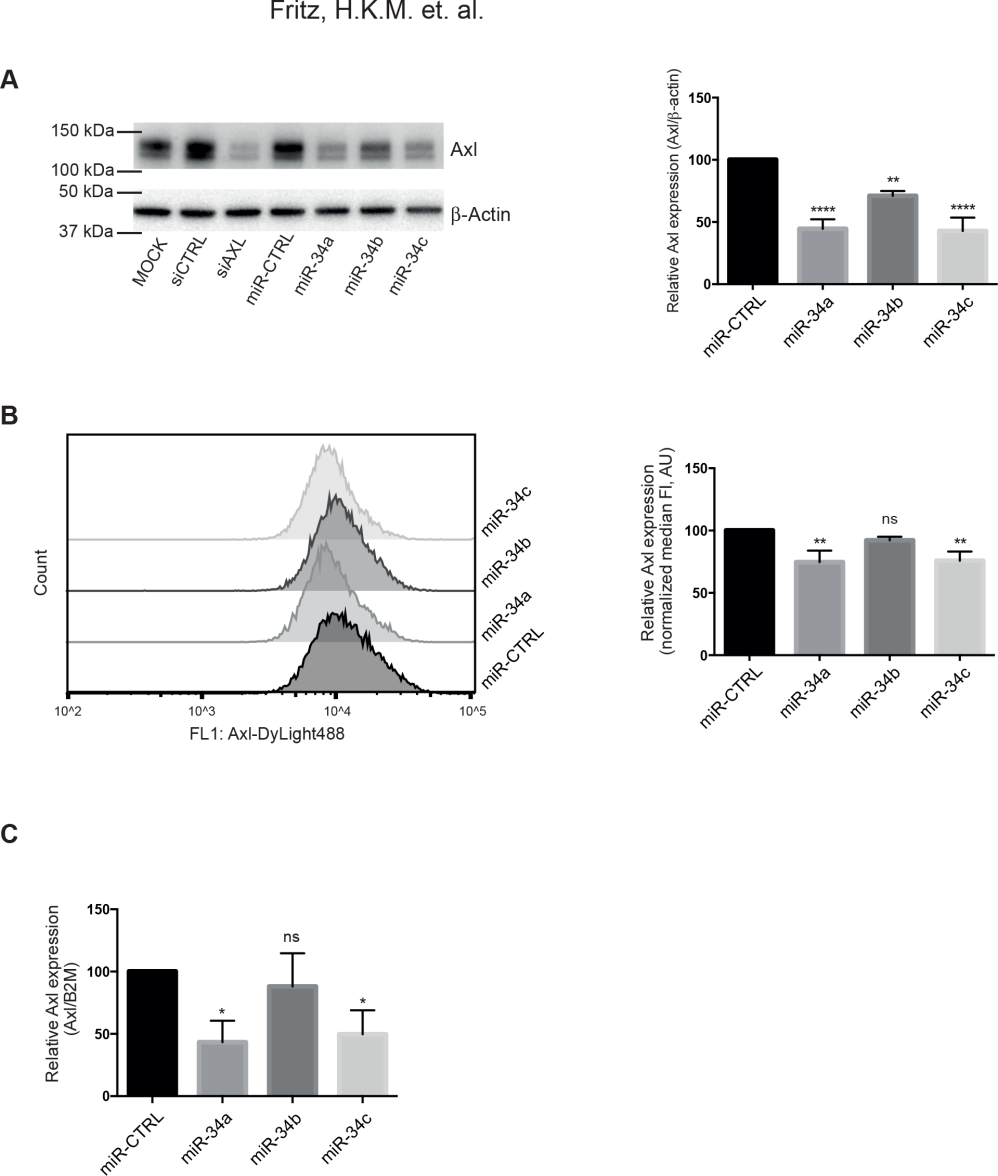
Decreased Axl expression in 786-O cells transfected with miR-34a/b/c. Cells were transiently transfected with miR-34a, miR-34b or miR-34c and assayed for Axl expression 72 hours after transfection. (**A**) Total cellular Axl was significantly decreased after transfection with all three miRNAs as judged by western blot (WB). Left panel, WB bands for Axl and β-Actin; right panel, quantification of the relative Axl WB band intensities. Axl siRNA and control siRNA were used as controls. (**B**) Axl surface expression was significantly decreased in cells transfected with miR-34a and miR-34c, as evaluated by flow cytometry. Left panel, representative flow cytometry image; right panel, quantification of the relative median fluorescence intensity. (**C**) RT-qPCR for Axl mRNA revealed that Axl mRNA levels are decreased in miR-34a- and miR-34c-transfected 786-O cells. Data presented as mean ±SD from three independent experiments. One-way ANOVA test with Dunnett’s multiple comparison test was used. ****, p < 0.0001; **, p < 0.01; *, p < 0.05.

We hypothesized that the Axl overexpression that has been observed in RCC could be due to decreased levels of miR-34a or the other two miR-34 family members. Therefore, we investigated whether transfection of 786-O cells with the miR-34 family members resulted in downregulation of Axl protein expression. Both miR-34a and miR-34c significantly downregulated Axl protein as judged by both WB (Fig 1A) and flow cytometry (Fig 1B). In miR-34a-transfected cells, Axl expression decreased to 45% as compared to control in both total cell lysates (Fig 1A) and to 75% at the cell surface (Fig 1B). Similarly, transfection with miR-34c caused a reduction of Axl expression to 43% in total cell lysates (Fig 1A), and to 76% on the cell surface (Fig 1B). In cells transfected with miR-34b, Axl was downregulated to 71% in total cell lysates (Fig 1A), but no significant reduction of cell surface Axl was observed when using flow cytometry (Fig 1B). A small but significant increase in Axl expression as determined by WB was observed in both siCTRL and miRNA-CTRL samples, as compared to MOCK cells. However, the downregulation of Axl by siAXL, as well as miR-34a or miR-34c, remained significant also when compared to MOCK-transfected cells.

### Decreased Axl mRNA levels in miR-34a transfected 786-O cells

As miRNAs can act through inhibition of translation or degradation of target mRNA transcripts, we determined if Axl mRNA levels were altered in miR-34a/b/c-transfected cells using RT-qPCR. In 786-O cells transfected with miR-34a, Axl mRNA was significantly decreased to 43% as compared to control (Fig 1C). Similarly, Axl mRNA levels were decreased to 50% in cells transfected with miR-34c (Fig 1C). In contrast, in cells transfected with miR-34b, no significant change in Axl mRNA levels could be detected (Fig 1C).

### miR-34a and miR-34c interact with the 3’ UTR of Axl mRNA in regulation of translation

**Fig 2:**
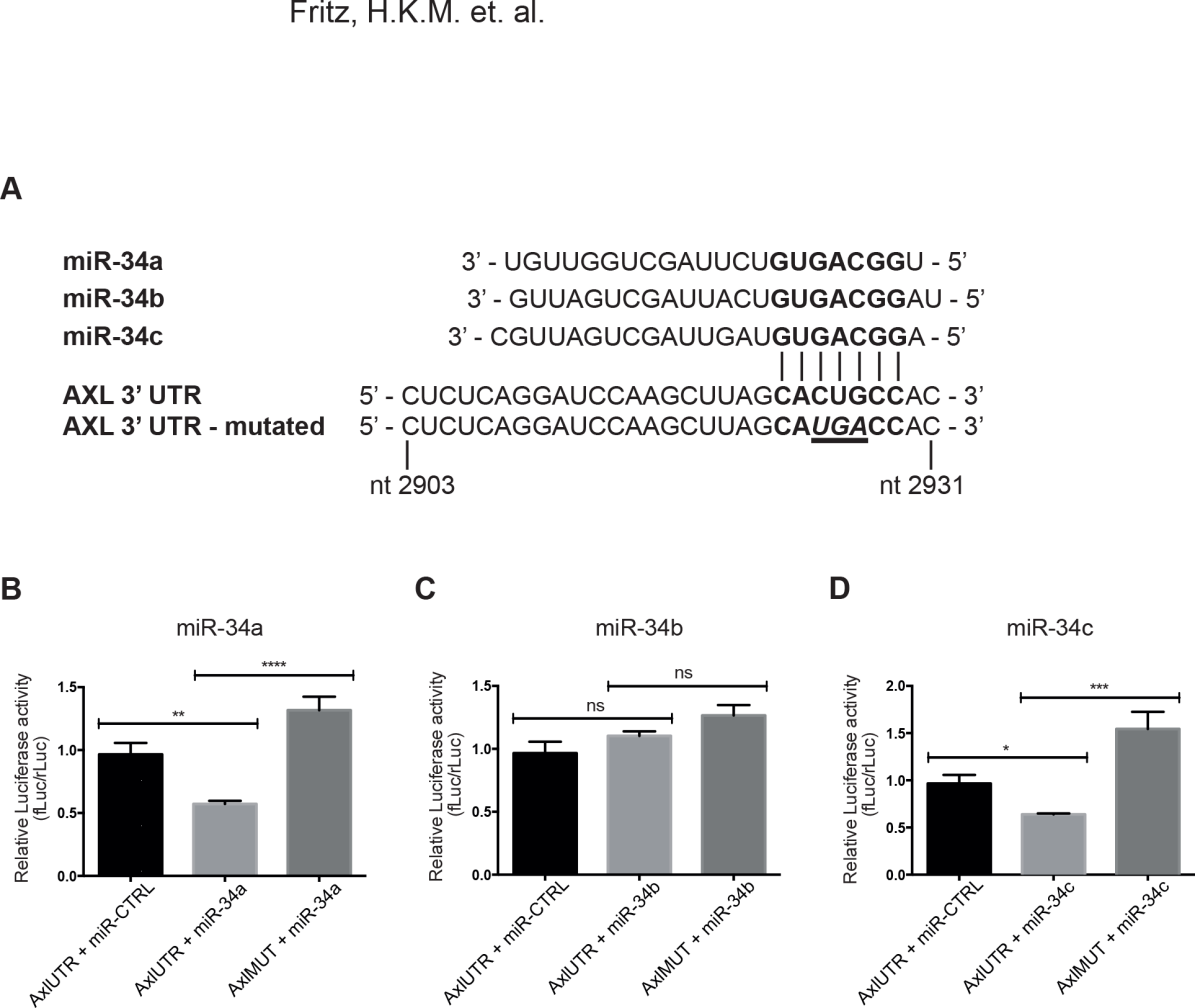
miR-34a and miR-34c interact with the Axl 3’UTR. The ability of the miR-34 family members to interact with and regulate Axl mRNA was evaluated using luciferase assay. HEK293 cells were transfected with a firefly luciferase reporter plasmid containing the Axl 3’UTR (pMirTarget-AxlUTR), an internal control Renilla luciferase plasmid, and the miRNA mimics or negative control miRNA mimic. Luciferase activity was measured 48 hours after transfection. Control experiments were performed with a reported plasmid where the 8-nt seed sequence in the Axl 3’UTR was mutated (pMirTarget-AxlMUT). (**A**) The miR-34a/b/c sequence complementarity to Axl 3’ UTR (shown in bold text), and the introduced mutation in the Axl 3 UTR seed sequence (shown in underlined bold italic text). Nucleotide positions are shown as nucleotide positions within the full Axl mRNA transcript (RefSeq: NM_021913.3) (**B**) Luciferase activity was significantly decreased in cells co-transfected with miR-34a and the pMirTarget-AxlUTR, as compared to cells co-tranfected with miRNA-CTRL and pMirTarget-AxlUTR or cells co-transfected with miR-34a and pMirTarget-AxMUT. (**C**) No significant decrease in luciferase signal was detected with miR-34b. (**D**) Luciferase activity was significantly decreased in HEK293 cells co-transfected with miR-34c and the pMirTarget-AxlUTR, as compared to control samples. Data from three independent experiments are presented as mean ±SD. One-way ANOVA test with Dunnett’s multiple comparison test was used. ****, p < 0.0001; ***, p < 0.001; **, p < 0.01; *, p < 0.05.

To investigate whether miR-34a/c regulate the expression of Axl by interaction with the 3’UTR of Axl mRNA, a Luciferase reporter assay was performed in HEK293 cells, which themselves do not express detectable Axl, and in addition the HEK293 cells express low levels of the miR-34 family members (S1 Fig). The miR-34a/b/c mimics and Axl 3’ UTR Firefly luciferase reporter plasmid (pMirTarget-AxlUTR) were co-transfected along with an internal control Renilla vector. The seed sequence region of pMirTarget-AxlUTR and pMirTarget-AxlMUT, as well as the miR-34 family members are shown in Fig 2A. Both miR-34a and miR-34c decreased the luciferase signal; the signal decreased 41% with miR-34a (Fig 2B), and 34% with miR-34c (Fig 2D), whereas miR-34b had no effect (Fig 2C). These results indicate that both miR-34a and miR-34c interact with the 3’ UTR of Axl mRNA. Moreover, co-transfection of a variant Axl 3’ UTR Firefly luciferase reporter plasmid carrying a mutation at the predicted binding site for miR-34a and miR-34c (pMirTarget-AxlMUT) with miR-34a (Fig 2B) or miR-34c (Fig 2D) resulted in an increased luciferase signal, as compared to results obtained with the pMirTarget-AxlUTR vector with the same miRNAs. This indicates that the observed downregulation in luciferase signal with miR-34a and miR-34c is due to binding of these miRNAs to the predicted target site in the Axl 3’ UTR. When miRNA-CTRL or no miRNA was used, no differences in luciferase signal were observed for wild-type or mutant pMirTarget-AxlUTR vectors (S2 Fig). However, it is noteworthy that the luciferase signal was significantly increased in cells transfected with pMirTarget-AxlMUT along with miR-34c as compared to those transfected with pMirTarget-AxlUTR along with miRNA-CTRL (Fig 2D).

### Lack of correlation between miR-34a and Axl mRNA in RCC tumor tissue

**Fig 3:**
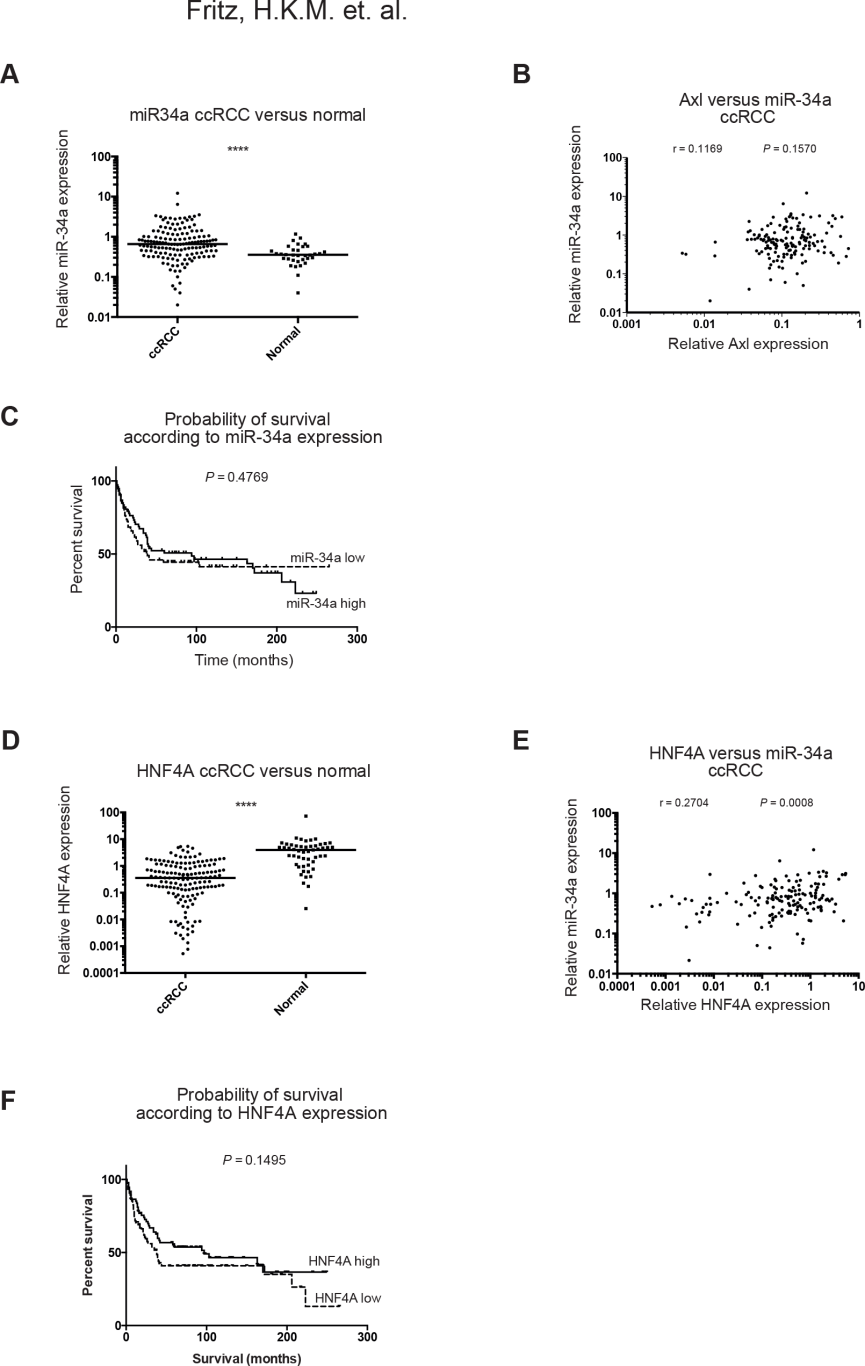
Expression of the miR-34 family, Axl and HNF4A in RCC. The levels of miR-34a, Axl mRNA, and HNF4A mRNA were determined using RT-qPCR in a cohort of RCC patients (cohort N = 198, ccRCC N = 152, normal N = 50). (**A**) Relative expression of miR-34a in ccRCC tumor samples and normal samples. A significant increase in miR-34a expression was found in ccRCC patients as compared to normal kidney tissue. The line marks the median in each group. (**B**) Spearman correlations for miR-34a versus Axl mRNA in ccRCC patients. No significant correlation was found. (**C**) Kaplan-Meier survival analysis for miR-34a in ccRCC patients, divided into two groups; the 50% of patients with miR-34a expression above median versus the 50% of patients with miR-34a expression below median. Log rank test revealed that miR-34a expression is not associated with patient survival in ccRCC. (**D**) Relative expression of HNF4A in ccRCC tumor samples and normal samples. HNF4A expression was decreased in ccRCC patients as compared to normal kidney tissue. The line marks the median in each group. (**E**) Spearman correlations for miR-34a versus HNF4A mRNA in ccRCC patients. A weak but significant positive correlation was found. (**F**) Kaplan-Meier survival analysis for HNF4A in ccRCC patients, divided into two groups; the 50% of patients with HNF4A expression above median versus the 50% of patients with HNF4A expression below median. Log rank test showed that HNF4A expression is not associated with patient survival in ccRCC. The miRNA levels were normalized to the geometric mean of U47, RNU44 and RNU48, whereas Axl and HNF4A mRNA were normalized to B2M mRNA. The Axl mRNA expression data used for comparison have previously been published [10]. ****, p < 0.0001; ***, p < 0.001; **, p < 0.01; *, p < 0.05.

To investigate whether Axl mRNA levels correlated with miR-34a or miR-34c expression in RCC tumors, RT-qPCR was used to measure the three miRNA-family members in RCC tumor tissue (N = 198), and in kidney cortex tissue (N = 50) and compared to Axl mRNA expression data from the same cohort, which has previously been reported [10] (Table 1). The miR-34a levels were significantly higher in ccRCC as compared to kidney cortex tissue (Fig 3A and S3 Fig). Spearman correlation analysis revealed no significant correlation between Axl and miR-34a expression in RCC patients (Fig 3B). Similarly, no significant correlation between Axl and miR-34a was found when analyzing the data in the publically available ccRCC cohort from The Cancer Genome Atlas (TCGA) (S4 Fig). However, for both miR-34b and miR-34c, a weak positive correlation was found (S3 Fig) in our cohort. Additional analyses for possible associations between expression of miR-34 family members and previously reported [10] Axl protein levels in patient serum and tumors (S1, S2 and S3 Tables) revealed no correlation. A weak inverse correlation was found between miR-34b and Axl mRNA in non-ccRCC patients (S1 Table), however our luciferase reporter assay indicates that Axl mRNA is not directly regulated by miR-34b (Fig 2B). It is noteworthy that expression levels for both miR-34b and miR-34c were very low in RCC patient samples, and we did therefore not follow up on this finding further.

**Table 1:** Cohort clinical characteristics.

Variable	Clear Cell RCC (ccRCC)	Papillary RCC (pRCC)	Chromophobe RCC (chRCC)	Oncocytoma
Patients, *n*	152	27	11	8
TNM stage, I+II/III/IV	66/38/48	14/7/6	5/4/2	N/A
Metastasis status, M0/M1	105/47	21/6	9/2	N/A
Nuclear grade, 1/2/3/4	8/33/77/34	4/9/11/3	0/2/8/1	N/A
Tumor size, mm range	20–170	25–180	30–150	30–100
Vein invasion, no/yes	94/57	20/7	7/4	6/0
Capsule formation, no/yes	104/45	17/8	8/2	3/0
Survival, median months (% 5-year survival)	42 (47.0)	51 (45.6)	N/A (81.8)	N/A (100.0)
Patient age, range years	36–85	25–82	36–80	50–80
Patient sex, male/female	87/65	17/10	4/7	4/4

Clinical characteristics of the patient cohort, divided into the renal cell carcinoma (RCC) tumor subtypes.

### Lack of evidence of a mutated miR-34a seed sequence within the Axl 3’ UTR

To determine whether the observed lack of negative correlation between miR-34a and Axl mRNA levels in ccRCC was explained by alterations in the seed sequence for miR-34a within the Axl 3’ UTR, we sequenced a randomly selected portion (N = 95) of the ccRCC tumor samples. All samples except one rendered sequencing data covering the region if interest. According to the sequencing analysis in the 94 ccRCC samples, the miR-34a seed sequence region within the Axl 3’ UTR was unaltered.

### Lack of association between miR-34-a/b/c levels and survival in ccRCC patients

Survival analysis was used to determine whether miR-34a expression affects survival in ccRCC patients. According to Kaplan-Meier analysis and Log Rank test there was no association between any of the tested miRNAs and survival (Fig 3C and S3 Fig).

### HNF4A is decreased in ccRCC but correlates positively with miR-34a

The lack of correlation between miR-34a and Axl expression levels led us to hypothesize that miR-34a could primarily act through regulation of other target mRNAs, such as the tumor suppressor HNF4A, which is a known target of miR-34a. To investigate whether HNF4A mRNA levels correlated with miR-34a expression in RCC tumors, HNF4A expression was measured in ccRCC tumor tissue (N = 152), and in normal kidney cortex tissue (N = 50) using RT-qPCR. HNF4A expression levels were significantly decreased in ccRCC, as compared to kidney cortex tissue (Fig 3D). However, miR-34a and HNF4A expression levels correlated positively rather than negatively (Fig 3E), suggesting that miR-34a does not act as a major negative regulator of HNF4A in ccRCC. Similarly, analysis for correlation between miR-34a and HNF4A in TCGA database on ccRCC did not yield support for negative regulation of HNF4A mRNA levels by miR-34a (S4 Fig). In our cohort, Kaplan-Meier survival analysis revealed no association between HNF4A expression and patient outcome in ccRCC patients (Fig 3F).

## Discussion

In this report, we demonstrate that transfection of 786-O cells with miR-34a and miR-34c resulted in decreased Axl expression. Using a luciferase reporter assay we confirmed that both miR-34a and miR-34c bind directly to the seed sequence in the 3’ UTR of the Axl mRNA transcript. These findings are in agreement with previously published reports regarding Axl and miR-34a in cancer [22, 31–33], and support the theory of miR-34a as a tumor suppressor also in RCC [23]. However, we found miR-34a to be upregulated in a large cohort of ccRCC patients, which is in agreement with reports from small sample sets [24, 34, 35]. In our large ccRCC patient cohort there was no correlation between miR-34a, or miR-34c, and the expression of Axl. We found no mutations or SNPs within the miR-34a seed sequence in the Axl 3’ UTR, indicating that the lack of correlation is not due to genetic alterations modulating the miR-34a binding site within the Axl mRNA transcript. Thus, we conclude that in ccRCC the levels of Axl are not primarily regulated by the miR-34a/c. However, it should be noted that tumor tissue lysates contain several different cell types in addition to tumor cells, such as fibroblasts and endothelial cells, possibly diluting or disguising any effects on Axl expression by miR-34a.

In our cohort of ccRCC patients we could not demonstrate any significant correlation between survival and the levels of any of the investigated miR-34 family members. This is not in support of an important role of these miRNAs as tumor suppressors. In this context, it is interesting to note that in a study with miR-34 knockout mice, neither of the miR-34 knockouts displayed increased tumorigenicity, and p53-mediated tumor suppression was found be intact [36]. Based on these results, the concept of the miR-34 family as important players in p53-mediated tumor suppression has been debated [37]. Results of an experimental rat kidney carcinogenesis model also argue against miR-34a playing a role in suppression of tumor growth. In this model, miR-34a was found to be upregulated and inhibition of miR-34a in the RCC cells resulted in suppressed proliferation [25]. Moreover, in a study conducted in B-cell lymphoid cells increasing miR-34a expression resulted in anti-apoptotic effects in cells overexpressing Myc [38]. These observations support the idea that the effects of miR-34a are highly cell type-dependent [25].

In our luciferase reporter assay experiments, we observed that the luciferase signal was increased by miR-34c in cells with pMirTarget-AxlMUT, as compared to those transfected with pMirTarget-AxlUTR and miRNA-CTRL. This could indicate that miR-34c possibly has another, yet unknown, low-affinity target site in the Axl 3’ UTR with opposite regulatory effects as compared to the seed sequence investigated in this report. Another possible explanation could be that miR-34c interacts with RNA-binding proteins or other regulatory RNAs, thereby indirectly regulating Axl 3’ UTR in a positive manner.

Our experimental data clearly shows that miR-34a and miR-34c have the capacity to regulate Axl expression in RCC cells. However, our results from primary tumors did not support the idea and rather suggest that the miR-34 family has little influence on Axl mRNA expression in ccRCC. In our previous report on Axl in RCC, we evaluated the tumor tissue for presence of Axl protein using immunohistochemistry and made the noteworthy observation that there was no correlation between the Axl mRNA levels and the immune staining intensity [10]. In the present investigation, we also did not find any association between the levels of miR-34 family members and the immune staining for Axl in tumor tissue, or soluble Axl in patient serum.

We also evaluated the expression of HNF4A mRNA in our cohort and found that HNF4A mRNA is decreased in ccRCC tumors, in agreement with previously reported findings [28]. HNF4A has been reported to be a tumor suppressor in hepatocytes, and loss of HNF4A is associated with increased proliferation, and increased tumor size and number in an induced hepatic tumor model [27]. However, in the ccRCC cohort, we found a weak positive correlation between miR-34a and HNF4A, suggesting that increased miR-34a levels cannot explain the observed downregulation of HNF4A in ccRCC. In this context, it is noteworthy that a SNP in one of the two target sequences for miR-34a within the HNF4A 3’ UTR has previously been reported in RCC [39], possibly explaining the lack of negative correlation between miR-34a and HNF4A.

For both miRNAs and siRNAs, the efficiency of target silencing has been shown to depend on the total concentration of available target transcripts [18]. Furthermore, it has been shown that miRNAs regulate threshold levels of mRNAs below which repression of translation occurs very efficiently even with modest targeting by the miRNA, and inversely that high target abundance could saturate the miRNA pool [40]. Considering that the abundance of mRNA target influences repression efficiency [18], and the high expression levels of Axl in RCC, it is possible that Axl mRNA transcripts are too abundant for repression by endogenous miR-34a. Another explanation could be that other target transcripts are thermodynamically favored, and therefore are primary targets of miR-34a in ccRCC, considering that target repression is a complex mechanism dependent on several factors including secondary structures, seed sequence matching and proximity to other regulatory elements, as well as interactions with other miRNAs [41, 42].

Taken together, our findings show that although Axl can be regulated by miR-34a and miR-34c *in vitro*, these miRNAs are not the primary regulators of Axl *in vivo* in ccRCC. In addition, we have demonstrated that miR-34a expression is increased in ccRCC but does not correlate with patient outcome, indicating that miR-34a does not function as a tumor suppressor in ccRCC.

## Supporting Information

S1 Fig (A) Basal Axl protein in 786-O and HEK293 cells as determined by WB. (B) Axl mRNA levels in 786-O and HEK293 cells, measured using RT-qPCR and normalized to B2M expression. (C) Basal expression levels of miR-34a, miR-34b and miR-34c in 786-O and HEK293 cells, as determined using RT-qPCR and normalized to RNU48 expression. Student’s *t* test (N = 3).
(EPS)

S2 Fig (A) Representative image showing flow cytometry gating and controls used for Axl staining. (B) Controls used in the luciferase assay. No significant difference in luciferase signal could be detected between the controls.
(EPS)

S3 Fig (A) Pairwise comparison of miR-34a (left), miR-34b (middle) and miR-34c (right) expression in 45 matched pairs of ccRCC and kidney cortex tissue. Wilcoxon matched-pairs signed rank test. (B) Relative expression of miR-34a (left), miR-34b (middle) and miR-34c (right) expression in ccRCC tumor subtypes and normal kidney cortex samples. The line marks the median in each group. (C) Spearman correlations for miR-34b (left) and miR-34c (right) versus Axl mRNA in ccRCC patients. (C) Kaplan-Meier survival analysis for miR-34b (left) and miR-34c (right) in ccRCC patients, divided into two groups; the 50% of patients with miR expression above median versus the 50% of patients with miR expression below median. (EPS)

S4 Fig Level 3 RNA-seq data containing normalized miR expression values were downloaded from The Cancer Genome Atlas (TCGA) data portal (http://tcga-data.nci.nih.gov/tcga/dataAccessMatrix.htm) by March 2013. The data comprised 284 ccRCCs analyzed on the Illumina Genome Analyzer (GA) platform and 217 ccRCCs analyzed on the Illumina HiSeq platform. Pearson correlation analysis was performed on logged normalized expression values on miR-34a, Axl and HNF4A within the two data sets using the R software (http://cran.r-project.org).
(EPS)

S5 Fig Original blots for Axl and β-actin expression in 786-O cells as shown in Fig 1. The blotting procedure has been described in the Materials and Methods section.
(TIF)

S1 Table Spearman correlations for miR-34a/b/c versus Axl mRNA levels in different RCC patient categories. (DOCX)

S2 Table Spearman correlations for miR-34a/b/c expression levels versus Axl protein in patient serum, as determined by ELISA[10], in different RCC patient categories. (DOCX)

S3 Table Kruskal-Wallis test for miR-34a/b/c expression levels versus Axl protein in patient tumors, as determined by immunohistochemistry (IHC, data from [10]), in different RCC patient categories. Axl IHC staining was scored and categorized as negative, low, or high, as described [10].
(DOCX)
