# Supplementary figures and images for: The Axl-Regulating Tumor Suppressor miR-34a Is Increased in ccRCC but Does Not Correlate with Axl mRNA or Axl Protein Levels
Source: PLoS One. 2015 Aug 19;10(8):e0135991. doi: 10.1371/journal.pone.0135991 (PMC4546115; doi:10.1371/journal.pone.0135991)

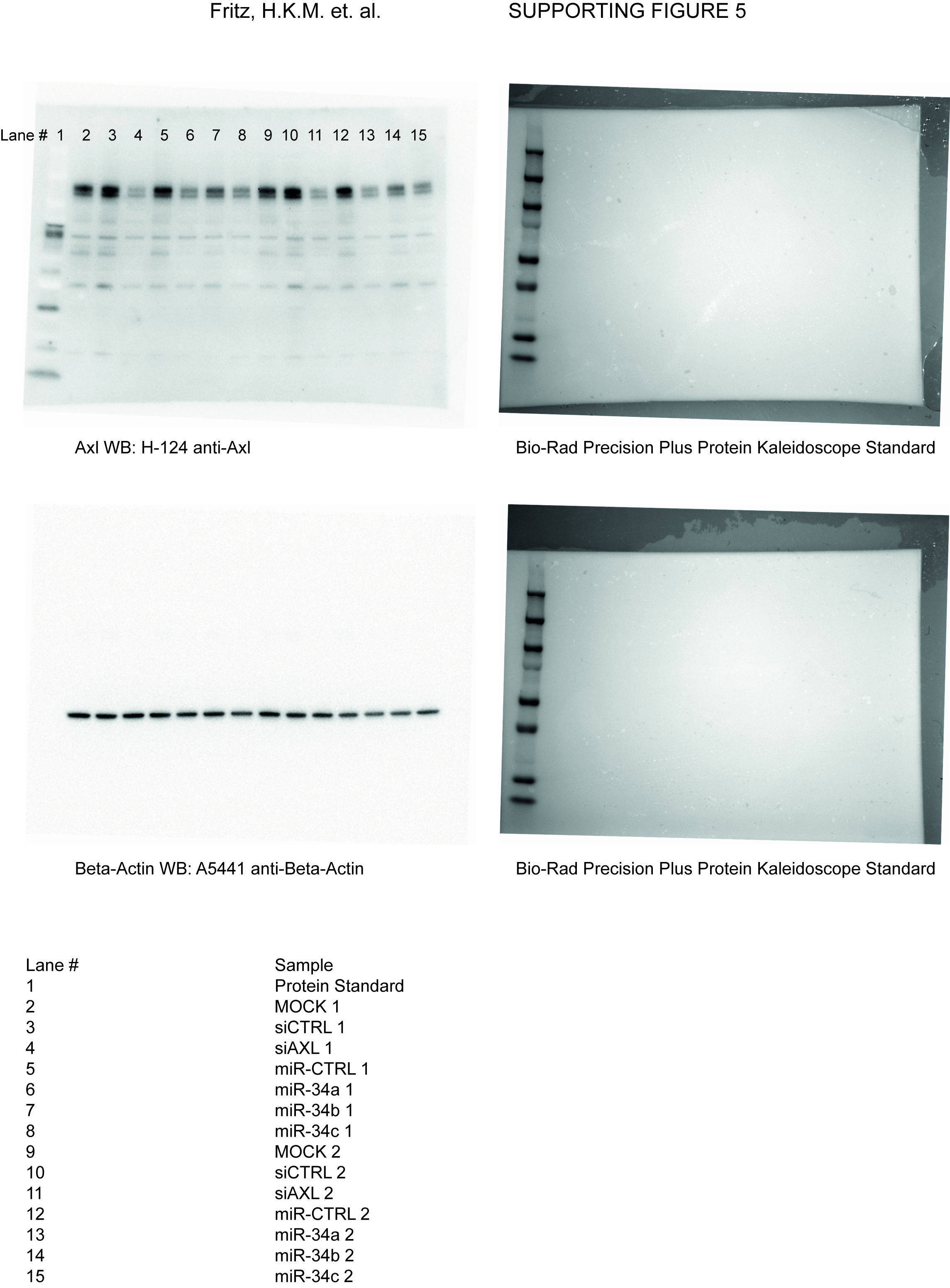

Supplement: S5 Fig — The blotting procedure has been described in the Materials and Methods section. (TIF) [file pone.0135991.s005.tif]
