# Supplementary material for: The Axl-Regulating Tumor Suppressor miR-34a Is Increased in ccRCC but Does Not Correlate with Axl mRNA or Axl Protein Levels
Source: PLoS One. 2015 Aug 19;10(8):e0135991. doi: 10.1371/journal.pone.0135991 (PMC4546115; doi:10.1371/journal.pone.0135991)
Supplement: S1 Table — (DOCX) [file pone.0135991.s006.docx]

| **microRNA** | **Patient category** | **Spearman r** | ***P* value** |
| --- | --- | --- | --- |
| miR-34a | All RCC | 0.09235 | 0.2026 |
| miR-34a | Non-ccRCC | -0.01621 | 0.9168 |
| miR-34b | All RCC | 0.04522 | 0.5334 |
| miR-34b | Non-ccRCC | -0.3982 | 0.0074 |
| miR-34c | All RCC | 0.05818 | 0.4228 |
| miR-34c | Non-ccRCC | -0.2750 | 0.0708 |

**Supporting Table 1.** Spearman correlations for miR-34a/b/c versus Axl mRNA levels in different RCC patient categories.
