# Supplementary material for: The Axl-Regulating Tumor Suppressor miR-34a Is Increased in ccRCC but Does Not Correlate with Axl mRNA or Axl Protein Levels
Source: PLoS One. 2015 Aug 19;10(8):e0135991. doi: 10.1371/journal.pone.0135991 (PMC4546115; doi:10.1371/journal.pone.0135991)
Supplement: S2 Table — (DOCX) [file pone.0135991.s007.docx]

| **microRNA** | **Patient category** | **Spearman r** | ***P* value** |
| --- | --- | --- | --- |
| miR-34a | All RCC | -0006116 | 0.9363 |
| miR-34a | ccRCC | -0.02892 | 0.7410 |
| miR-34b | All RCC | 0.008178 | 0.9150 |
| miR-34b | ccRCC | -0.01875 | 0.8304 |
| miR-34c | All RCC | 0.07448 | 0.3301 |
| miR-34c | ccRCC | 0.07454 | 0.3938 |

**Supporting Table 2.** Spearman correlations for miR-34a/b/c expression levels versus Axl protein in patient serum, as determined by ELISA, in different RCC patient categories.
