# Supplementary material for: The Axl-Regulating Tumor Suppressor miR-34a Is Increased in ccRCC but Does Not Correlate with Axl mRNA or Axl Protein Levels
Source: PLoS One. 2015 Aug 19;10(8):e0135991. doi: 10.1371/journal.pone.0135991 (PMC4546115; doi:10.1371/journal.pone.0135991)
Supplement: S3 Table — Axl IHC staining was scored and categorized as negative, low, or high, as described [10]. (DOCX) [file pone.0135991.s008.docx]

| **microRNA** | **Patient category** | **Kruskal-Wallis *P*** |
| --- | --- | --- |
| miR-34a | All RCC | 0.3493 |
| miR-34a | ccRCC | 0.4378 |
| miR-34b | All RCC | 0.7179 |
| miR-34b | ccRCC | 0.9325 |
| miR-34c | All RCC | 0.9938 |
| miR-34c | ccRCC | 0.8754 |

**Supporting Table 3.** Kruskal-Wallis test for miR-34a/b/c expression levels versus Axl protein in patient tumors, as determined by immunohistochemistry (IHC), in different RCC patient categories. Axl IHC staining was scored and categorized as negative, low, or high.
